# Effects of Various Antifouling Coatings and Fouling on Marine Sonar Performance

**DOI:** 10.3390/polym11040663

**Published:** 2019-04-11

**Authors:** Bradley Donnelly, Ian Bedwell, Jim Dimas, Andrew Scardino, Youhong Tang, Karl Sammut

**Affiliations:** 1 Centre for Maritime Engineering, Control and Imaging, College of Science and Engineering, Flinders University, South Australia 5042, Australia; bradley.donnelly@flinders.edu.au; 2 Maritime Division, Defence Science & Technology, Victoria 3207, Australia; Jim.Dimas@dst.defence.gov.au; 3 Defence Mission Systems, Thales Australia, New South Wales 2116, Australia; Ian.Bedwell@thalesgroup.com.au; 4 Institute for NanoScale Science and Technology, College of Science and Engineering, Flinders University, South Australia 5042, Australia

**Keywords:** fouling, acoustic sensors, antifouling coatings, transmission loss

## Abstract

There is a rising imperative to increase the operational availability of maritime vessels by extending the time between full docking cycles. To achieve operational efficacy, maritime vessels must remain clear of biological growth. Such growth can cause significant increases in frictional drag, thereby reducing speed, range and fuel efficiency and decreasing the sensitivity of acoustic sensors. The impact that various stages of fouling have on acoustic equipment is unclear. It is also unclear to what extent antifouling techniques interfere with the transmission of acoustic signals. In this study, to examine this effect, neoprene samples were coated with three antifouling coatings, namely, Intersmooth 7460HS, HempaGuard X7 and Hempasil X3. Other neoprene samples were left uncoated but were imbedded with the biocide, 4,5-dichloro-2-octyl-4-isothiazolin-3-one (DCOIT) during the mixing and curing process. Uncoated nitrile samples that had varying levels of fouling from immersion in Port Phillip Bay, Australia, for 92, 156 and 239 days were also extracted. The acoustic properties of these samples were measured using an acoustic insertion loss test and compared to uncoated neoprene or nitrile to ascertain the acoustic effects of the applications of antifouling coatings as well as the fouling growth itself. A T-peel test was performed on all coated samples in an attempt to understand the adhesive properties of the coatings when applied to neoprene. It was found that the application of antifouling coatings had little effect on the transmission characteristics of the neoprene with approximately 1 dB loss. The embedment of DCOIT, however, has a chance of causing aeration in the neoprene, which can heavily hamper transmission. An assessment of the effect of the fouling growth found that light and medium fouling levels produced little transmission loss, whereas more extreme fouling lead to a 9 dB transmission loss. The adhesion properties of the coatings were investigated but not fully ascertained as tensile yielding occurred before peeling. However, various failure modes are presented and discussed in this study.

## 1. Introduction

Fouling is the accumulation of unwanted substances, such as proteins molecules and organisms, on marine infrastructure such as pylons, boats or pipes due to exposure to their environment [1,2]. As fouling accumulates, it can have many adverse effects including increasing drag, reducing the maximum speed of a ship and increasing fuel consumption, weakening supports on oil rigs and reducing the functionality of many sensors [3]. Fouling can also cause environmental damage as fouled vessels can act as vectors for introducing invasive species into vulnerable environments [4,5].

The effect that fouling has on an acoustic signal can be undesirable, with signal degradation being measured as high as 3 and 5.5 dB for immersions of 200 and 300 days respectively [6]. Additionally, various forms of hard calcareous fouling, such as barnacles, have been recorded producing noise as they move around in their carapace [7]. Very little has been done to directly measure the effect that antifouling coatings have on the surfaces on which they are applied. Fouling-release coatings are some of the most commonly used technologies. These coatings have low surface free energy, which reduces the adhesion strength of some fouling organisms. Some low surface free energy coatings may also result in Cassie-Baxter wetting, which traps air bubble on the surface and could have a significant negative impact on acoustic functionality. One study, however, found that some fouling-release coatings have little or no effect on acoustic sensors, [8] but they do not clearly depict how this was ascertained. Many acoustic sensors are also acoustic projectors, and there is some indications that the emission of certain acoustic frequencies can inhibit fouling growth [9]. This phenomenon could be utilized on active sensors but on passive sensors, and sensors operating outside the key frequencies, it would be ineffective. 

Marine engineers are currently faced with a dilemma in regard to acoustic sensor performance. If the acoustic sensor is left uncoated, it will initially perform better than a coated surface. However, an uncoated surface will eventually foul and likely suffer from performance degradation. If the surface is coated, there will likely be an initial loss in performance due to the coating. However, if fouling protection is maintained for long periods, then extended performance of the sensor can be realized. A similar dilemma exists when treating optical sensors, a reduction in transparency in exchange for antifouling protection [10]. A key consideration is how long it would to take for fouling growth to cause a greater drop in transmission loss than the application of an antifouling coating. This study tries to quantify the impact of the antifouling coatings on sensor performance and the impact of fouling on sensor performance.

## 2. Materials Preparation and Characterizations

### 2.1. Materials

Polychloroprene (neoprene) and the curative zinc oxide were purchased from Romar Engineering, Sefton, NSW, Australia. The 4,5-dichloro-2-octyl-4-isothiazolin-3-one (DCOIT) was supplied by Dow Chemicals, Melbourne, VIC, Australia. Fouling-release coatings Hempasil X3 and HempaGuard X7 (containing copper pyrithione) were supplied by Hempel, Australia. A self-polishing copper oxide containing antifouling paint, Intersmooth 7460HS, was supplied by International Paints, Melbourne, VIC, Australia.

### 2.2. Sample Preparations

To create thin rubber panels, neoprene was mixed on a mill to soften the material. Once the neoprene was self-mixing, 3.1% *w*/*w*. zinc oxide was slowly added to the mill. To create 1 kg of the DCOIT embedded panels, neoprene was mixed on a mill at 45 °C, then 135 g of DCOIT was added slowly, and finally 27 g of zinc oxide. A total of 16 large panels were cured in 250 mm × 250 mm × 10 mm molds, 10 small panels were cured in 150 mm × 150 mm × 3 mm molds and 4 DCOIT embedded panels were cured in 250 mm × 250 mm × 30 mm molds at 150 °C for 50 minutes at a pressure of 20 MPa. Three additional panels, which were left uncoated, were also made as a control. The DCOIT panels were made thicker as they would affect the bulk modulus of the neoprene as well as its interfacial features. The neoprene panels were first cleaned and etched with 6% trichloroisocyanuric acid and ethyl acetate. Then, they were coated with a single coat of Nexus X-tend, followed by two coatings of either Hemapsil X3 or HempaGuard X7. For the International Paint samples, a single coat of the tie coat Intergard 276 was applied followed by three coats of Intersmooth 7460HS. After each coating was applied, the panels were rotated 90° to increase adhesive strength and left to dry overnight.

Additionally, 8 nitrile panels were made as substrates for fouling growth. Nitrile was selected for this as it conforms with the elastomers that are normally in use at DST (Defence Science and Technology). To create these panels, raw nitrile was mixed in a cold mill with 16% curatives w/w until fully mixed, then cured in 300 mm × 150 mm × 10 mm molds at 140 °C for 60 min. Six of these panels were deployed at a depth of 1–2 meters in temperate water in Port Philip Bay, Australia, on 18/06/2018 and 2 were not immersed and used as controls. To allow for different stages of growth, 2 light, medium and heavily fouled panels were recovered on 17/09/2018 (92 days), 20/11/2018 (156 days) and 11/02/2019 (239 days), respectively.

### 2.3. Material Surface and Mechanical Characterisation

Contact angle and density: The contact angle and density of the treated and untreated neoprene samples were examined to ascertain features that pertain to their acoustic and field performance. 

Goniometer: The surface energy of the neoprene samples was ascertained, as interfaces with low surface energy can experience Cassie-Baxter wetting [11,12]. Cassie-Baxter wetting is a major issue for acoustic transmission as it traps air bubbles on the surface which acts like a mirror for reflecting sound. It is also a design feature that has been associated with preventing fouling [13,14]. 

A KSV CAM200 goniometer (KSV, Helsinki, Finland) was used to measure contact angle at seven different points using the sessile drop method. The surface energy was determined using the equation of state theory [15] which utilises the thermodynamic relationship of interfacial tension given by Equation (1)

(1) $$\gamma_{sl}=\gamma_{l}+\gamma_{s}-2\sqrt{\gamma_{l}\times \gamma_{l}}\times e^{-\beta {\left(\gamma_{l}-\gamma_{s}\right)}^{2}}$$

To solve the Young’s equation given by Equation (2)

(2) $$\gamma_{s}=\gamma_{sl}+\gamma_{l}\;\times cos\theta$$

where $\gamma_{sl}$,$\;\gamma_{l}$,$\;\gamma_{s}$ are the surface tensions between: the substrate and the liquid, the liquid and air, and the substrate and air, respectively; cosθ is the contact angle; and β is a experimentally determined constant equal to 0.0001247. It should be noted that this calculation was performed by the measurement instrument.

Density: The density of each rubber sample was determined by comparing the dry weight against the submerged weight of each sample.

Fouling characterization: Based on ASTM D6990 and ASTM D3623–78a (2012), the fouling on the nitrile panels was characterized by the fouling rating (FR) system developed by the US Navy, as shown in Table 1. It is an established way to classify the amount and type of fouling present on a surface [16]. This considers the amount and type of fouling present and rates it based on classifications.

**Figure 1:**
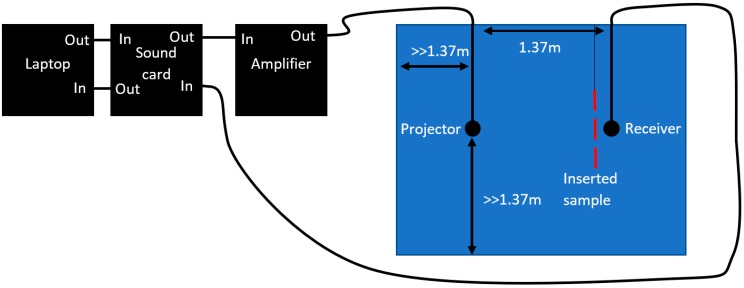
Insertion loss test setup.

Acoustic transparency characterization: To ascertain acoustic properties of each of the panels, an insertion loss test was performed [17]. To do this, the acoustic energy transmitted in the empty channel is measured. Then, a sample panel is inserted into the channel and the transmitted acoustic energy is measured again. The energy transmission loss is given by Equation (3)

(3) $$W_{t}=\;W_{c}-W_{s}$$

where $W_{c}$ is the energy transmitted in the empty channel and $W_{s}$ is the energy transmitted through the sample. Figure 1 shows the setup used for the insertion loss test. Two hydrophones (Teledyne Reson TC4033) were placed 1.37 m apart and used as both projector and source. A maximum length sequence (MLS) signal of pseudorandom noise was generated in MATLAB and interfaced by a Roland Octa Capture sound card to a Lab Gruppen 14,000 FP amplifier. The received signal was processed by the same card and cross-correlated with the transmitted signal to produce an impulse response of the system. This response was time gated to avoid reflections from the surface and tank walls. A fast Fourier transform was performed on the impulse response to obtain the frequency response. A frequency response was measured for both an empty channel and a channel with a sample panel placed directly in front of, but not touching, the receiver hydrophone. Subtraction of the sample frequency response from the empty channel reference frequency response cancels out all the effects from shared components, assuming they do not vary greatly. This leaves behind the transmission loss caused by the sample panel. To achieve the best results, two measurements need to be taken quickly enough to ensure that the testing conditions remain the same which maintains the assumption of unvarying components but also ensuring the reverberations of the previous measurement have dispersed. This highlights the importance of appropriate timing when completing experiments to mitigate the effects of compounding components.

Due to the transmit response of the Reson TC4033, the frequency responses were measured from 1–100 kHz. Uncoated neoprene samples were compared to the antifouling-coated samples.

Adhesion: As it is postulated that long-term acoustic performance is dependent on the surface staying free of fouling, it is important to make sure that the antifouling coating is appropriately adhered to the surface.

**Figure 2:**
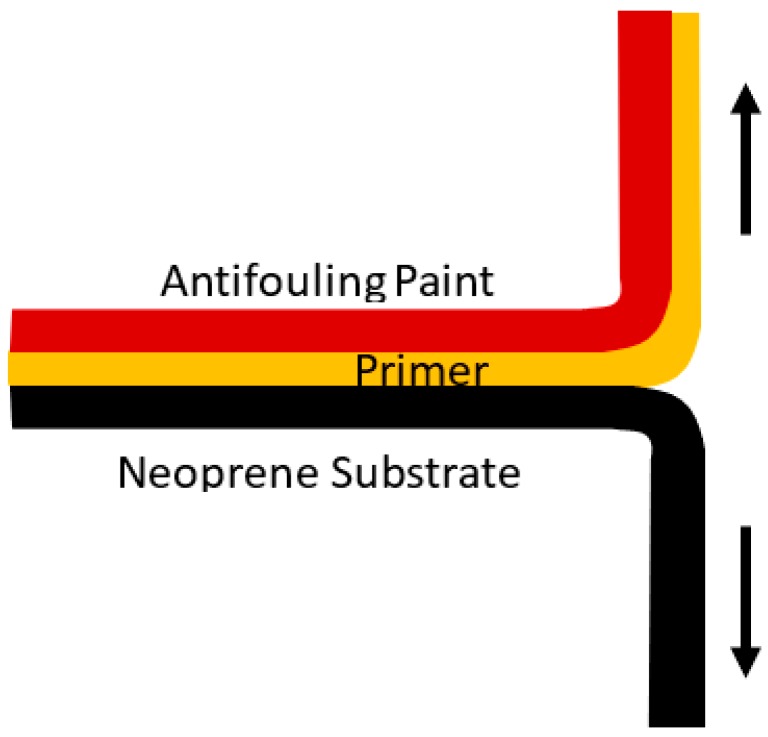
T-peel Test configuration.

To get a better understanding of the adhesion strength of the antifouling coatings, a T-peel test was performed. The method used was based on ASTM D1876 standard [18]. The 250 mm × 25 mm × 10 mm neoprene strips were prepared as before. Each of them was then etched with 3%, 6% or 12% trichloroisocyanuric acid and ethyl acetate. Each of the strips had the top 75 mm masked off by tape. One-third of the strips were coated with the Hemapsil X3 system (as described in Section 2.2), the second third were coated with the HempaGuard X7 system, and the final third were coated with the Intersmooth 7460HS system. After the coatings had dried, the masked portion was peeled back and inserted into the upper clamp of a universal testing machine (Shimadzu Scientific Instruments, Sydney, NSW, Australia) and the uncoated portion of the neoprene substrate was place into the lower clamp. The clamps were then separated at a rate of 50 mm/min and the applied force and displacement were recorded, as shown in Figure 2.

## 3. Results and Discussion

### 3.1. Material Surface and Sample Characterizations

The correspondence between the acoustic impedance of a transmission medium and that of the surrounding water is a key determiner for determining the sound transmission through the medium. The acoustic impedance is defined as the product of the speed of sound and the density. If there is an impedance mismatch, the thickness of the medium also contributes to transmission loss. The speed of sound in a medium, $v_{M}$ is related to both the density and the compressional modulus by Equation (4)

(4) $$v_{M}=\;\sqrt{\frac{K_{M}}{\rho_{M}}}$$

where $K_{M}$ is the compressional modulus and $\rho_{M}$ is the density, i.e., uncoated neoprene 1.34 g/cm^3^ and DCOIT neoprene 1.41 g/cm^3^. For rubbery materials, the compressional modulus is close to the bulk modulus of 3.22 MPa. The theoretical speed of sound in the neoprene is 1550 m/s and in the DCOIT-doped neoprene, it is 1551 m/s. It should be noted that the bulk modulus value was assumed based on common values for neoprene and may not be applicable to the DCOIT-doped neoprene. The main reason for this was to obtain an ideal sound speed in the neoprene, a more appropriate complex value will be discussed later in this study.

Using the framework described by Kinsler [19] and Morse, Ingard and Shankland [20], the theoretical transmission loss was determined. Employing the assumption that the transmitted wave is a plane wave and is traveling perpendicular to the normal of the panel, the panel was modelled as an infinite fluid layer with uniform thickness, L=0.01 m. To incorporate the attenuation, an approximate value of $1\times 10^{-4}f$ dB/m proportional to frequency was chosen. From this, a complex wave number k, bulk modulus K and sound speed c can be determined by the following Equations (5)–(7).

(5) $$k=\frac{\omega }{c_{0}}-j\times 10^{-4}f$$

(6) $$K=\;\rho {\left(\frac{\omega }{k}\right)}^{2}$$

(7) $$c=\;\sqrt{\frac{K}{\rho }}$$

where $c_{0}$ is the ideal speed of sound, ω is the angular frequency and f is the linear frequency.

**Figure 3:**
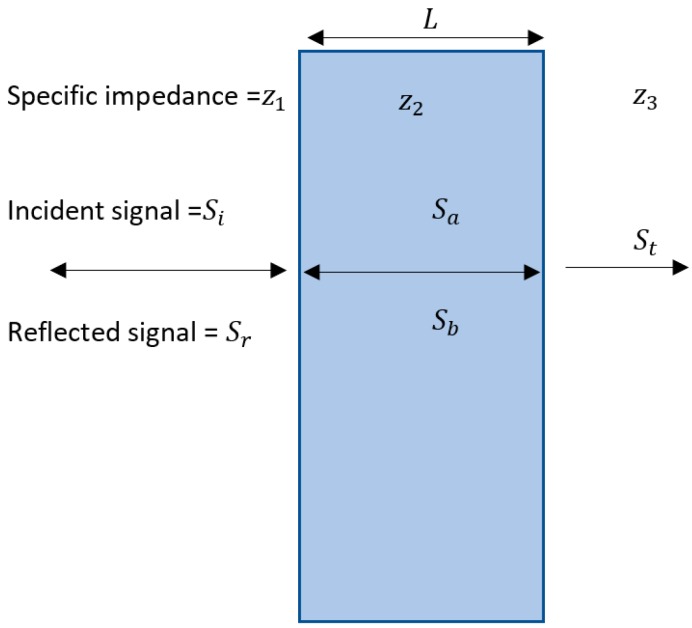
Transmission loss model.

Figure 3 shows the transmission loss model. In this model, there are two boundaries, the boundary where the signal enters the panel and the boundary where the signal exits the panel. At each of these, there are also two boundary conditions, the continuity of pressure and the continuity of particle velocity. The simultaneous equations that result from these conditions can be reduced to give the transmission coefficient [18,19], as shown below.

(8) $$T=\;\frac{\left(z_{3}+z_{1}\right)\cos\left(k_{2}L\right)}{2z_{3}}+\frac{j\left(z_{2}{}^{2}+z_{1}z_{3}\right)\sin\left(k_{2}L\right)}{2z_{2}z_{3}}$$

where $z_{1}$ is the acoustic impedance of the medium containing original incident wave, $z_{2}$ is the acoustic impedance of sample panel and $z_{3}$ is the acoustic impedance of the final medium. There is a special case of this model where the incident and final medium are identical, like something suspended in water, resulting in both mediums have the same acoustic impedance, i.e., $z_{1}=z_{3}$. This equality reduces Equation (8) to 

(9) $$T=\cos\left(k_{2}L\right)+\;\frac{j\left(\frac{z_{2}}{z_{1}}+\frac{z_{1}}{z_{2}}\right)\sin\left(k_{2}L\right)}{2}$$

The energy transmission loss in dB is given by Equation (10)

(10) $$TL=10log_{10}\left(T^{2}\right)$$

**Figure 4:**
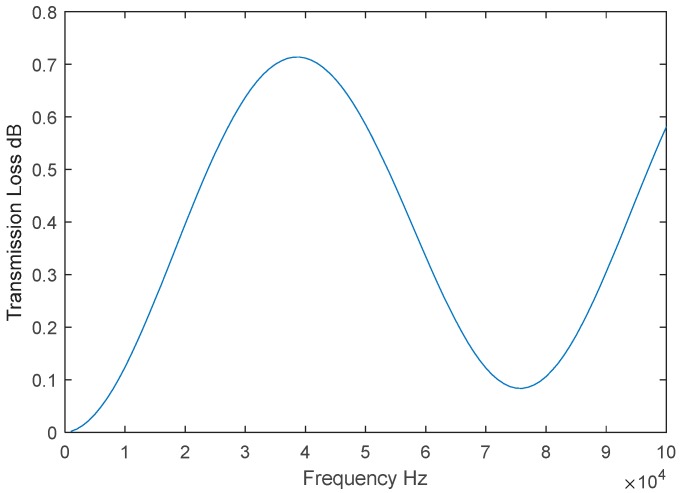
Transmission loss of uncoated 10-mm neoprene samples.

Based on these features, a theoretical transmission loss curve was generated and used to verify the results. As shown in Figure 4, the theoretical transmission loss is quite low. This is why neoprene was selected as the substrate.

### 3.2. Contact Angle and Surface Tension

**Figure 5:**
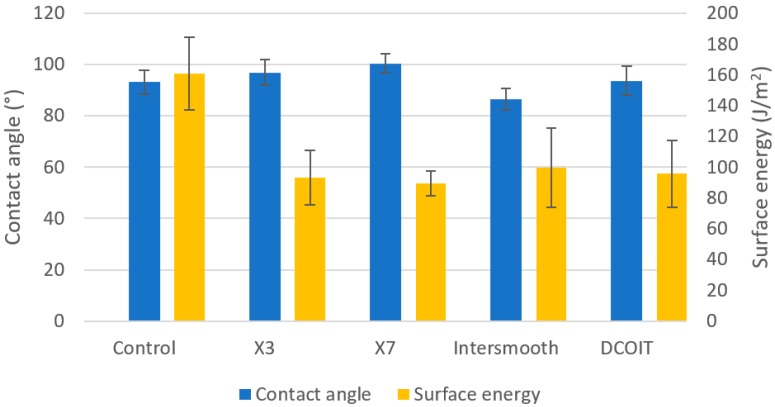
Average contact angle and surface energy.

As expected, and in accordance with the design requirements, both fouling-release coatings demonstrate lower surface energy and higher contact angles than the control samples. Although these features can lead to surface bubbles that could interfere with acoustic transmission, this effect was not observed in the samples tested. The DCOIT-doped rubber had a notably decreased surface energy, this is largely due to the hydrophobicity of the DCOIT, as shown in Figure 5. As no wetting artefacts were observed, it is unlikely that the hydrophobicity and surface energy has a significant effect on the acoustic transmission of the panels. However, the lower surface energies of all the treatments could have some impact on the antifouling efficacy.

### 3.3. Immersion

The nitrile samples were submerged for two years without antifouling protection and had a large range of fouling ratings present. As such, this material was used instead of neoprene as there was insufficient time to allow the neoprene samples to foul to a satisfactory level.

#### 3.3.1. Fouling Characterization

**Figure 6:**
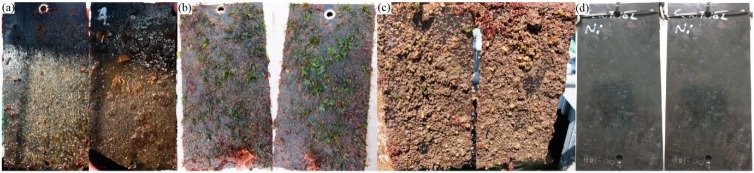
Nitrile samples with (**a**) light, (**b**) medium, (**c**) heavy, and (**d**) control fouling conditions.

Figure 6 shows the nitrile panels as they were recovered from Port Phillip Bay. The lightly fouled panels (6a) exhibited a large covering of slime but few or no larger algal fouling species and no hard calcareous fouling species, thus it was classified as FR 20 [16]. The medium fouled panels (6b) still contained a large slime covering, but also had extensive firmly attached green and red algae longer than a centimeter, thus it was classified as FR 30 [16]. The heavily fouled panels still contained some slime and weeds, but the majority of their surfaces were covered by a combination of calcareous fouling species, predominately barnacles slightly less than a centimeter across, thus they were classified as FR 70 [16]. These values are useful as they allow us to compare the three main stages of fouling, slime, weeds and hard fouling. FR 70 also represents the most common “heavy” fouling that will likely occur. Even though the fouling ratings can increase, many ship owners will attempt to clean a vessel’s hull at this point because of the already known increased running costs associated with heavy fouling.

#### 3.3.2. Acoustic Transparency Characterization

Each time a sample was tested, it would produce a frequency response $H{\left(f\right)}_{full\;channelX}$. This incorporates features of the transmitter, receiver, sample, coating and channel itself (plus noise). To extract the features that were caused by the coating, Equation (11) was used.

(11) $$H{\left(f\right)}_{coating}=H{\left(f\right)}_{full\;channel\;coated}-H{\left(f\right)}_{full\;channel\;bare\;sample}$$

This works on the assumption that everything in $H{\left(f\right)}_{full\;channel\;coated}$ is the same as $H{\left(f\right)}_{full\;channel\;bare\;sample}$ except for the coatings and they cancel each other out.

**Figure 7:**
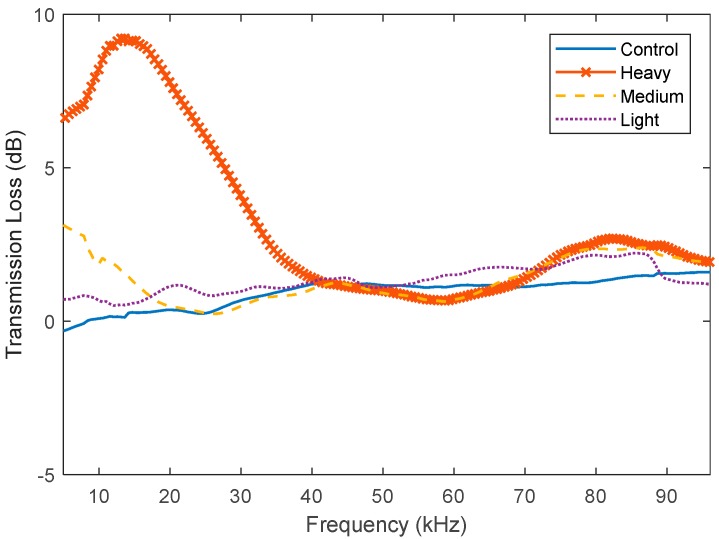
Acoustic transmission loss due to fouling growth on nitrile samples.

Figure 7 and Table 2 demonstrate that, as the level of fouling increases, so does the associated transmission loss. Interestingly, the largest transmission loss occurs at the lower frequencies, 5–33 kHz. Compared to the research by Fitzgerald [6], who found that the average transmission loss at 24.6 kHz after 300 days was 5.5 dB, the transmission loss obtained here at 24.6 kHz is 6.14 dB. The difference between these values could be due to many reasons such as the different species of fouling present and the different methods for measuring the fouling level (days immersed vs. FR). The above observations indicate that the transmission loss is highly dependent on the type of fouling present as well as the amount present. It also suggests that the acoustic transmission is not linearly related to the FR value. This is probably because the FR classification system is not intended to characterize the acoustic transmission properties as a function of fouling growth and also because large variations in acoustic properties can happen as fouling changes from soft bodies to hard calcareous types.

**Figure 8:**
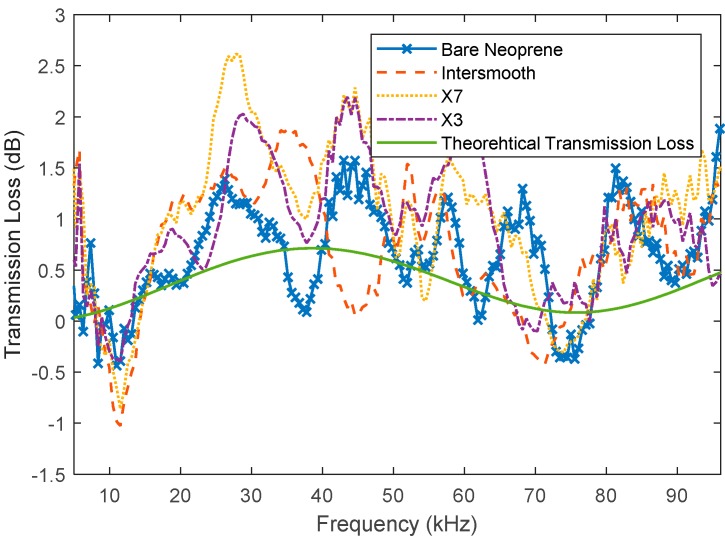
Acoustic transmission loss of uncoated neoprene and neoprene coated with Hemapsil X3, HempaGuard X7 and Intersmooth 7460HS.

**Figure 9:**
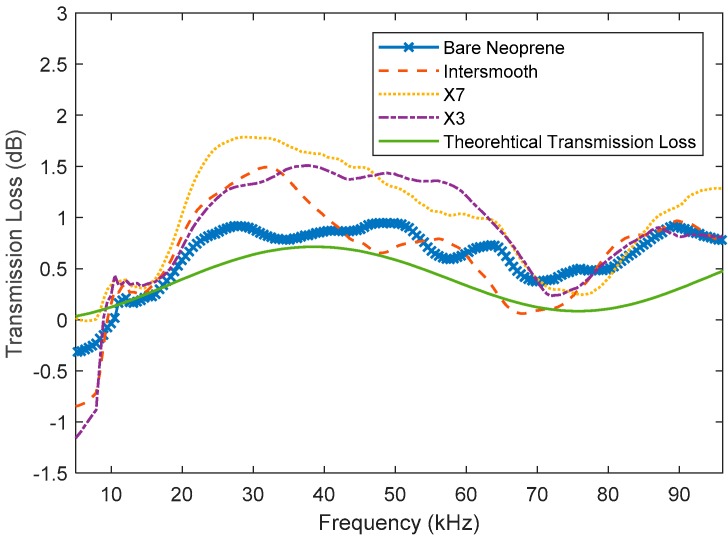
Smoothed acoustic transmission loss of uncoated neoprene and neoprene coated with Hemapsil X3, HempaGuard X7 and Intersmooth 7460HS.

Each of the antifouling-coated samples was compared to the uncoated neoprene for transmission loss. The raw data and smoothed data (moving mean filter) are depicted in Figure 8 and Figure 9 respectively. Here, it can be seen that the measured transmission losses have trends that are a similar shape to the theoretical transmission loss. It can also be seen that, as expected, the uncoated neoprene had the lowest measured transmission loss in general. Interestingly, it seems that the neoprene coated with Intersmooth 7460HS has slightly lower transmission loss than the Hempasil X3 and HempaGuard X7. However, none of the trends for the coated neoprene are more than 1 dB larger than the trends for the uncoated neoprene. 

#### 3.3.3. Comparison of the Effects of Coating and Fouling

By comparing the X7 and X3, it appears that there is little difference between their responses. They both also have higher measured surface energy. X7 also contains copper pyrithione and Intersmooth 7460HS contains copper oxide, with a slightly better response seen from the Intersmooth 7460HS samples. This suggests that the surface energy plays a larger role in acoustic transmission response than the bulk features such as copper loading. It is likely that this would change with coating thickness, as bulk features would become more exaggerated and interfacial features remain constant as thickness increases. By comparing the results presented in Table 2 and Table 3, it can be seen that, across the board, the changes in acoustic properties caused by the growth of fouling is greater than the changes due to the application of antifouling coatings. 

**Figure 10:**
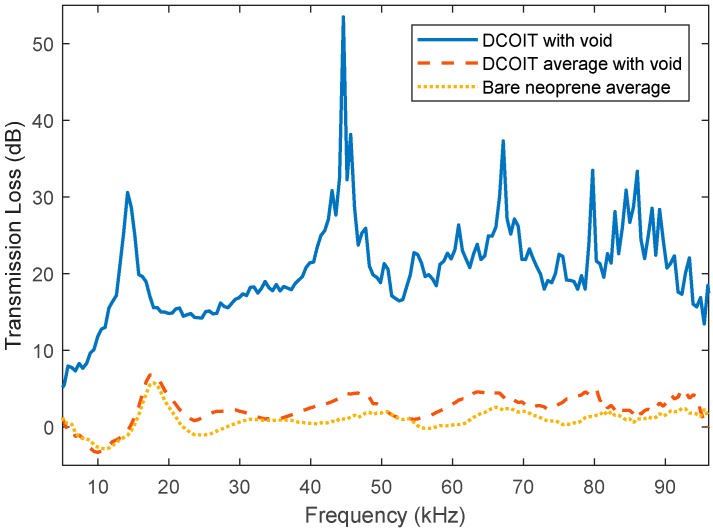
Average acoustic transmission loss of bare neoprene and DCOIT-doped neoprene.

The measured average transmission loss of the DCOIT samples showed a large increase in transmission loss compared to the average of uncoated neoprene. An inspection of the transmission loss of each DCOIT sample clearly revealed that one sample was skewing the average. Figure 10 shows the average DCOIT, without the skewing data points, compared to the average uncoated neoprene and highlights the extent to which one sample was quite different. This shows that correct DCOIT samples have a transmission loss very similar to that of uncoated neoprene. However, there is some risk of defects occurring. The postulated reason for the defect causing the large difference in transmission loss is due to a void that formed during the curing process. The void is most likely due to the thermal instability of the DCOIT which can expand at temperatures close to the neoprene curing temperature. While the DCOIT itself has only a small effect on the acoustic transmission characteristic, it does result in an increased chance of bubbles being created in the neoprene matrix. This suggests that DCOIT would be a suitable antifouling solution provided that appropriate quality assurance measures are in place during the application and curing process to ascertain when bubbles form. This could probably be performed using ultrasonic equipment and techniques similar to those used to measure coating thickness. 

### 3.4. Adhesion

The results of the peel test in regard to the determination of the adhesion strength of each coating were not clear cut. In most cases, the coating yielded to the applied tension before any peeling occurred. Table 4 shows a breakdown of the results.

**Figure 11:**
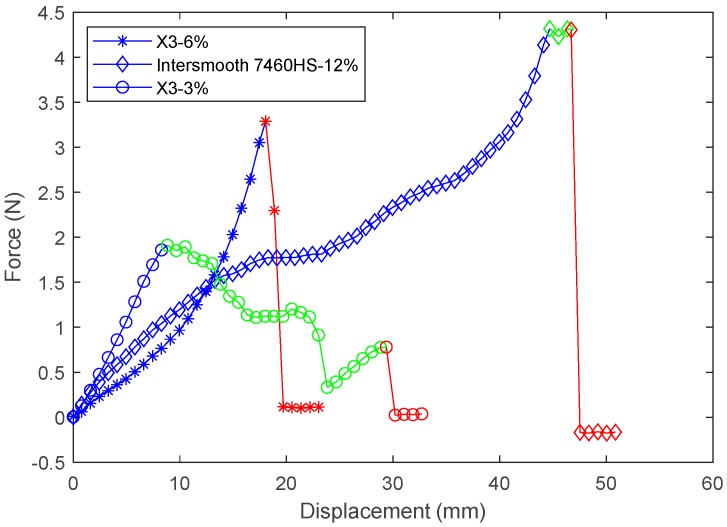
Examples of peeling modes.

These results suggest that the surface preparation of the substrate could affect the adhesion strength. Peeling occurred on all coatings with a 3% etchant strength preparation. For the 6 and 12% etchant strength preparation, there were mixed results. Overall the average peak force was higher with the Intersmooth 7460HS coatings than with either of the Hempasil X3 and HempaGuard X7 fouling-release coatings regardless of etchant strength, as shown in Table 3. To improve the results of this test, the method could be adjusted so that a vertically symmetrical system can be used as opposed to pulling directly on the coating itself. This would allow the applied force to be high enough to cause adhesive failure without tensile failure occurring. The results from the peel test indicate the only statement that adhesion strength of the antifouling coatings is greater than their tensile strength. Figure 11 shows some of the failure modes that occurred during these tests [21]. In each figure, the blue section shows the initial deformation of the coating, the green section shows where peeling occurred, and the red section shows when the coating yielded to the tensile stress. 

The failure mode of an X3-6% response, represented by asterisks, could be classed as structural failure, this is where the coating is broken at the start of the bonding surface. This mode of failure was the most common observed. It is a result of the bonding strength of the coating being greater than its tensile strength. The failure mode present shown by the response of an Intersmooth 7460HS-12%, represented by diamonds, is an example of adhesive failure. This is the classic sort of peeling where the adhesive bond fails, and the coating and the substrate delaminate. This only occurred for a short time, however, and it peeled along its entire width which is demonstrated by the fact that the final peak is the same height as the initial peak, suggesting the cross-section area didn’t change. This was the most common peeling mode that occurred, appearing in all systems apart from the X3-3%. The response for the X3-3%, represented by circles, is characteristic of mixed modes of failure occurring. In the peeling section adhesive as well as structural failure is occurring. The cohesive failure is evident due to the large negative trend of the peeling section plus the lower final yield peak compared to the initial one, suggesting a decrease in the cross-sectional area. Adhesive failure is evident due to the large gap between the initial peak and the final yield peak, showing that the clamps moved a further 20 mm apart, which would only be possible if peeling occurred. This was the rarest peeling phenomena that occurred, only appearing on the Intersmooth 7460HS-3% and X3-3% systems. It also appears to be the least desirable mode, as the peel length was longer than the purely adhesive failure in all cases. The fact that these occurred in the 3% strength etchant systems suggests that etching plays an important role in the adhesion strength.

## 4. Conclusions

This study has demonstrated the importance of fouling to acoustic sensor performance. Heavy fouling produced a large transmission loss of up to 9 dB, while light and medium fouling had a small effect but indicated a relationship between fouling growth and acoustic performance. The study also indicated that the FR system may be unsuited for predicting acoustic properties. Features like biofilm thickness and average organism size, while more difficult to obtain, may produce clearer relationships. More data points, however, would be needed to support this theory. In contrast, there was little effect of the antifouling coating choice to protect the underlying rubber substrate. A non-biocidal fouling-release coating, a fouling-release coating containing copper pyrithione and a traditional self-polishing antifouling coating containing copper oxide all produced very little transmission loss compared to the uncoated rubber substrate, generally less than 1 dB. However, the results suggest that interfacial features of a coating play a larger part in their acoustic properties than their bulk features. This is likely due to the fact that coatings are designed to be applied relatively thin (<1 mm), which de-emphasizes the effect that bulk features have. Therefore, marine engineers should pay particular attention to choosing the optimal antifouling coating to provide the longest-lasting fouling protection to the sensor based on time and location of deployment and the ability of the coating to adhere to the underlying substrate for prolonged marine immersion. They also need to consider the interfacial components of the coating to be applied. Compared to other marine surfaces, where hydrophobicity is an important feature, when considering acoustic surfaces, we need to ensure that sufficient wetting occurs. Although proper wetting is important to achieve good transmission, it is secondary to the expected antifouling efficacy of the coating. Doing these things will ensure optimal sensor performance as well as reduce weight, drag, flow noise and maintenance costs through the life of the platform.

## Figures and Tables

**Table 1:** Fouling rating (FR) definitions.

Type	Fouling Rating	Description
Soft	0	A clean, foul free surface.
Soft	10	Light shades of red and green incipient slime. Bare metal visible beneath the fouling.
Soft	20	Slime as dark green patches with yellow or brown colored areas (advanced slime). Bare metal is obscured by the fouling. **Fouling can be removed by hand.**
Soft	30	Green weed as filaments up to 75 mm in length, projections up to 6 mm in height or a flat network of filaments that are green, yellow or brown in color. Soft non-calcareous fouling projecting up to 6 mm in height. **The fouling cannot be easily removed by hand.**
Hard	40	Calcareous fouling in the form of encrusting bryozoans **less than** 6 mm in height.
Hard	50	Calcareous fouling in the form of tubeworms **or** barnacles **less than** 6 mm in diameter or height.
Hard	60	**Combination** of encrusting bryozoans, tubeworms or barnacles, **less than** 6 mm in diameter or height.
Hard	70	**Combination** of encrusting bryozoans, tubeworms or barnacles, **greater than** 6 mm in diameter or height.
Hard	80	Tubeworms closely packed together and growing upright away from the surface. Barnacles, tubeworms and/or encrusting bryozoans growing one on top of another, 6 mm **or less** in height. Calcareous shells appear clean or white in color.
Hard	90	Dense growth of tubeworms, encrusting bryozoans or barnacles, 6 mm **or greater** in height. Calcareous shells brown in color or with slime or grassy overlay.
Composite	100	All forms of fouling present, particularly soft sedentary animals without calcareous covering growing over various forms of hard growth.

**Table 2:** Comparison of change in transmission loss due to fouling with respect to control.

Coating	Peak Difference (dB)	Average Difference (dB)	5–33 kHz Average Difference (dB)	33–65 kHz Average Difference (dB)	65–100 kHz Average Difference (dB)
Light (FR 20)	1.92	0.43 ± 0.38	0.69 ± 0.39	0.17 ± 0.18	0.42 ± 0.38
Medium (FR30)	4.28	0.55 ± 1.03	1.28 ± 1.53	−0.23 ± 0.14	0.55 ± 0.84
Heavy (FR70)	9.09	2.49 ± 3.17	6.40 ± 2.0	−0.02 ± 0.49	2.06 ± 2.88

**Table 3:** Comparison of change in transmission loss due to coating with respect to bare neoprene.

Coating	Peak Difference (dB)	Average Difference (dB)	5–33 kHz Average Difference (dB)	33–65 kHz Average Difference (dB)	65–100 kHz Average Difference (dB)
X7	0.97	0.38 ± 0.31	0.52 ± 0.29	0.51 ± 0.21	0.31 ± 0.32
X3	−1.34	0.20 ± 0.38	−0.03 ± 0.55	0.54 ± 0.14	0.17 ± 0.29
Intersmooth	0.68	0.02 ± 0.30	0.09 ± 0.41	−0.01 ± 0.27	0.01 ± 0.27

**Table 4:** Results from the coating peel test.

Coating–Etchant Strength	Average Peak Force (N)	Did Peeling Occur?	Average Peel Force (N)	Average Peel Length (mm)
7460HS-3%	5.76 ± 0.33	Yes	5.50 ± 0.81	45.36 ± 0
7460HS-6%	4.70 ± 0.08	No	N/A	N/A
7460HS-12%	4.46 ± 0.31	Yes	3.50 ± 0.27	4.63 ± 2.61
X7-3%	3.48 ± 0.49	Yes	3.27 ± 0.11	0.69 ± 0.18
X7-6%	3.35 ± 0.80	No	N/A	N/A
X7-12%	3.67 ± 0.47	No	N/A	N/A
X3-3%	3.21 ± 0.55	Yes	1.14 ± 0.46	20.53 ± 0
X3-6%	3.74 ± 0.32	No	N/A	N/A
X3-12%	3.32 ± 1.01	No	N/A	N/A
